# Discussion on Secure Standard Network of Sensors Powered by Microbial Fuel Cells

**DOI:** 10.3390/s23198227

**Published:** 2023-10-03

**Authors:** Helbert da Rocha, Paolo Caruso, João Pereira, Pedro Serra, Antonio Espirito Santo

**Affiliations:** 1 Department of Electromechanical Engineering, University of Beira Interior, 6200-001 Covilhã, Portugal; joao.luis.pereira@ubi.pt (J.P.); pmserra@ubi.pt (P.S.); aes@ubi.pt (A.E.S.); 2 Instituto de Telecomunicações, Delegação da Covilhã, 1049-001 Lisboa, Portugal; 3 Department of Industrial Engineering, University of Salerno, 84084 Fisciano, Italy; pcaruso@unisa.it

**Keywords:** energy harvesting, low-power security, IEEE 1451 family of standards

## Abstract

Everyday tasks use sensors to monitor and provide information about processes in different scenarios, such as monitoring devices in manufacturing or homes. Sensors need to communicate, with or without wires, while providing secure information. Power can be derived from various energy sources, such as batteries, electrical power grids, and energy harvesting. Energy harvesting is a promising way to provide a sustainable and renewable source to power sensors by scavenging and converting energy from ambient energy sources. However, low energy is harvested through these methods. Therefore, it is becoming a challenge to design and deploy wireless sensor networks while ensuring the sensors have enough power to perform their tasks and communicate with each other through careful management and optimization, matching energy supply with demand. For this reason, data cryptography and authentication are needed to protect sensor communication. This paper studies how energy harvested with microbial fuel cells can be employed in algorithms used in data protection during sensor communication.

## 1. Introduction

The interconnection of sensors maximizes the availability of information gathered from the process and enhances decision-making capabilities [1]. Using standards enables different entities to interconnect their products, thus contributing to system interoperability [2]. The IEEE 1451 standard defines the necessary aspects for sensor and actuator interconnection within a network. Alongside defining the network components and their internal structure, the standard outlines communication protocols [3].

Radio communications enable the placement of sensors in remote and hard-to-access locations, operating on battery power. However, the combination of site and battery power presents a challenge. While batteries require periodic replacement, placing sensors in specific locations can make this task difficult [4,5].

Energy harvesting presents an efficient and appealing alternative for powering radio-linked sensors. Scavenging energy from the sensor’s operating environment makes sustaining the device’s operation possible. However, energy collection must be carried out using methodologies that ensure optimal performance of the energy transducer [4].

Typically, the amount of energy harvested is limited, and energy availability can swing. Therefore, it is essential to implement operating mechanisms within the smart sensor that account for its energy usage while considering the energy requirements of the sensor itself.

The Internet of Things (IoT) security is a fundamental challenge. Optimizing the energy consumption of your sensors with cryptography is a challenging but important task, as cryptography can protect data from unauthorized access or modification but consumes more energy for computation and communication. According to Aslan et al. based on the 2020 Unit 42 IoT Threat Report, an estimated 98% of the IoT traffic is not encrypted [6]. Energy management must precisely communicate the sensors’ security needs with energy constraints. The security in energy-harvesting networks has been ignored [7].

This work proposes a solution that fulfills the requirements mentioned above. The approach described herein discusses the implementation of a secure low-energy sensor network compliant with the IEEE 1451 family of standards, wherein smart sensors are powered by microbial fuel cells (MFCs). Following this introduction, the subsequent sections will highlight the motivation for undertaking this work. The introductory section concludes by acknowledging the innovative nature of the proposed approach.

### 1.1. Motivation

The growing use of sensors and the increasing demand for their interconnection to enhance efficiency have led to the development of standards to accelerate the interoperability of diverse elements created by different entities. However, to ensure the success of these solutions, a set of challenges must be addressed. Energy availability stands at the core of these challenges, as the compromised operation of a sensor node arises in the absence of sufficient energy to ensure secure communication during the messages exchange.

The assessment of various energy sources available in the sensor’s operational environment, along with the efficiency of the transducer responsible for converting that energy (whether thermal, kinetic, electromagnetic, or chemical) into electrical energy, becomes essential. Optimizing the performance of the energy transducer, which converts non-electrical forms of energy into electrical power, is of utmost importance. Maximizing energy collection and storage capabilities also necessitates establishing low-consumption operations within the sensor. Furthermore, it is crucial to recognize that energy production capacity is not constant but varies over time. Consequently, energy availability within the node is subject to fluctuations and depends on collection capacity and the tasks assigned to the node.

Implementing energy management mechanisms becomes imperative to ensure efficient operation within the sensor network. These mechanisms aim to regulate the utilization of energy available within the node, considering the dynamic nature of energy availability and the specific requirements of the assigned tasks.

By addressing these energy-related challenges, a sensor network can operate effectively and maintain a reliable energy supply, enabling seamless interconnection and facilitating the overarching goal of increased efficiency in sensor-based systems [8].

### 1.2. Novelty

The development of wireless sensor networks, based on a standard that uses energy collected from the environment, allows for the expansion of the lifetime of the sensor elements. In any case, it is necessary to have operating mechanisms that would enable operation compatible with the IEEE 1451 standards. This work presents a discussion on how to combine security based on authentication and cryptography with a sensor network powered by an MFC. The energy produced by the reactor is regulated and stored for use by the sensor node. Based on the state of the art, the energy consumption of the cryptography algorithm was analyzed and discussed to secure communication using energy harvesting.

Previous works using the IEEE 1451 family of standards implementation were analyzed to provide information for implementing the node following the IEEE 1451.0 standard [9,10]. The structure used for the performance is the one defined for an Interface Module Transducer (TIM). A Power Management System (PMS) manages available power. The sensor’s energetic efficiency is improved by making use of sleeping modes. The TIMs are interconnected with the user’s network via the Network Capable Application Processor (NCAP). This network element is responsible for managing the functioning and interface of the TIMs with the user application [11].

### 1.3. Contribution

The discussion herein proposed relates to the need to promote approaches capable of developing real-world solutions, bridging the gap between different knowledge areas. Creating a sensor network, as established by the IoT, depends on making that network wireless. The power requirements of sensor nodes must be met. Harvesting energy at the operation site is one possible solution. Following a standard is paramount for real-world applications. This article deals with each one of these subjects.

The previously described scenario is conceptualized in this article by an MFC-powered sensor. The sensor implementation follows the IEEE 1451 standard. The standard infrastructure uses an NCAP through which each TIM connects to the user’s network. These two elements are wirelessly connected. Even though the standard supports WiFi, Bluetooth, ZigBee and 6LowPan, known for their strong communication capabilities, the energetic restrictions force wireless communications without any communication protocol. Accordingly, all the description and authentication procedures are processor tasks [12].

This work will clarify and reinforce the need for integrated solutions while highlighting the importance of including protection mechanisms between NCAPs and TIMs.

The remainder of this paper presents the IEEE 1451 family of standards in Section 2. Section 3 presents the energy-harvesting process using MFC. The security algorithms are presented in Section 4, focusing on the security of low-power sensors. Section 5 discusses the energy the MFC generates with security for low-power transducers. The conclusions are presented in Section 6.

## 2. Standard Network of Sensors

The IEEE 1451 family of standards comprises a leading standard, the IEEE 1451.0-2007 [13]. This standard defines an intelligent interface for transducers (sensors and/or actuators). It describes the TIM that contains the transducer connected to it. Each transducer and TIM has a Transducer Electronic Data Sheet (TEDS). The TEDS stores information about the transducer inside the TIM or outside of the TIM as a Virtual TEDS. There are four mandatory TEDS: Meta TEDS, TransducerChannel TEDS, User’sTransducerName TEDS, and Physical TEDS. The Meta TEDS stores information about the TIM, e.g., the manufacturer, the physical location where it was assembled, the number of transducer channels, and the control group. The TransducerChannel TEDS stores information about each transducer connected to the TIM, e.g., the channel ID, the transducer configuration, a timeout for data acquisition, an SI unit for conversion data from analog to digital and vice versa, and other information attached for the transducer. The User’s Transducer Name TEDS stores what the user wants to use to name the transducer inside the system. Moreover, the Physical TEDS stores information about radio and wired information to communicate the TIM to the NCAP.

**Figure 1:**
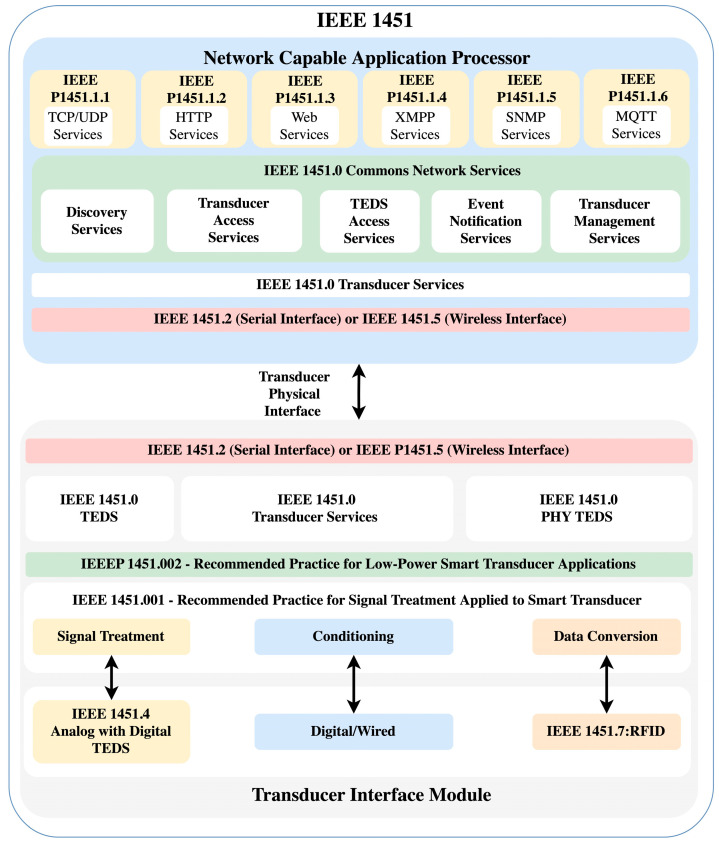
IEEE 1451 family of standards internal organization.

The NCAP started in early 2000 and focused on defining a common interface for a transducer as the IEEE 1451.1-1999 standard [14], bringing the concept of a common object model description for connecting the transducer to an external network. In 2007, this standard was merged with the IEEE 1451.0. The NCAP defines how the transducer can be connected to an internal network, receiving commands from an application and sending them to the TIM. The NCAP supports the following application protocols to communicate, e.g., HTTP, XMPP, SNMP, and MQTT. The IEEE 1451.0-2007 is under review as the IEEE P1451.0 standard. The IEEE 1451 family of standards is shown in Figure 1.

This family of standards has been used for the development and communication of transducers. The author in [15] developed a diagnostic, prognostic, and maintenance of corrosion for military aircraft and rotorcraft based on developing and testing different sensors with energy-harvesting techniques to parameterize the corrosion environment and predict its maintenance by laboratory testing. The IEEE 1451 family of standards was employed to develop and communicate with the sensor network developed using the low-power MSP 430 Microcontroller (MCU) from Texas Instruments (TI). Kumar and Hancke developed a low-power system to control and monitor indoor environments in real time based on parameters such as humidity, temperature, CO, and CO_2_ based on the ASHRAE55-2013, ISO7730 and IEEE 1451 family of standards [16]. Kumar et al. developed a more complex indoor monitoring system employing the exact conjunction of standards and the IEEE 1451.1 implemented in LabVIEW [17]. Jooste et al. implemented an autonomous irrigation scheduling system based on the low-power sensors and Zigbee connection employing the IEEE 21451 standards. The sensors measure the soil moisture measure, light, CO_2_, temperature, and humidity [18].

## 3. Energy Harvesting

### 3.1. Microbial Fuel Cells

The energy–water–food nexus presents significant challenges, particularly when addressing energy production or extraction from renewable sources. The ability to harness bioenergy by using biomass or taking advantage of naturally occurring processes is particularly interesting as a non-pollutant and renewable energy source.

**Figure 2:**
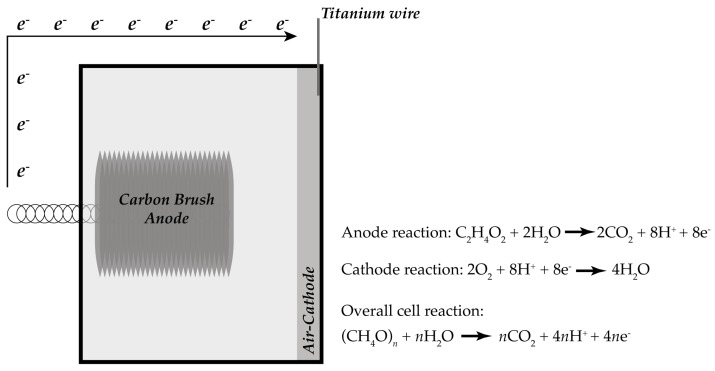
A single-chamber air-cathode MFC reactor representation, indicating the anode, cathode and overall cell reaction.

MFCs allow energy extraction from bacterial metabolism in an anaerobic environment. The absence of any common electron acceptor, like nitrate, sulfate or oxygen, is paramount to exploiting this energy. These fuel cells are very similar to common batteries, not needing to be recharged. MFCs, represented in Figure 2, are a specific type of the former, where bacteria use wastewater (fuel) to produce electrons. In anaerobic conditions and providing an oxygen source in a separate chamber, bacteria digest the fuel and exhibit an exoelectrogenic behavior: electrons are made available outside the bacteria cell wall. When the two electrodes from the MFC (as in a generic fuel cell) are connected, these electrons migrate from the anode (low redox potential) to the cathode (high redox potential), producing a current [19].

Simple substrates, e.g., acetate and glucose, were used in the initial experiments on MFCs to minimize the variables involved. Acetate is a direct fuel for the Krebs cycle (also known as the citric acid cycle) and does not allow any other respiratory pathway for the microbes at room temperature. It is also the end product of several metabolic processes. Acetate-based MFCs produced more than twice the maximum electric power of protein-rich wastewater. Glucose was less efficient: acetate converted 42% of its energy, and glucose converted only 3% [20].

Microbial communities that are not well suited for MFCs and the production of methane are the main challenges for generating electricity with MFCs using real wastewater [21]. The characteristics of real wastewater are extremely diverse, varying in pH, temperature, nutrient and chemical composition—which can include microplastics, antibiotics or other toxic compounds [22,23]. Even seasonality impacts the wastewater composition: wastewater in the summer is more concentrated than in the winter [24,25]. To overcome these issues and continue exploring how this technology can be improved, the use of acetate and a stabilized version of the reactor have been standardized [26].

**Figure 3:**
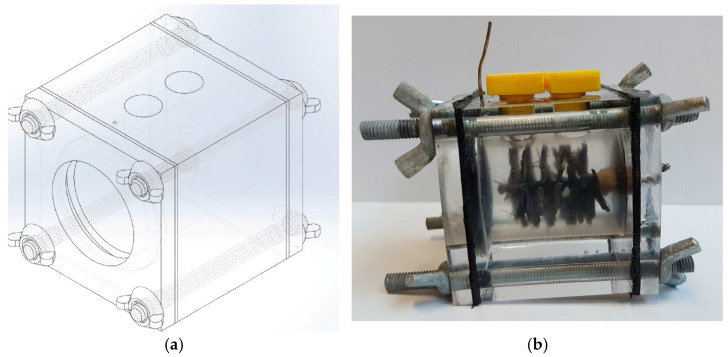
A 3D illustration and a single-chamber air-cathode MFC reactor. (**a**) Three-dimensional (3D) representation, (**b**) Another 3D representation.

The reactor, pictured in Figure 3, is a cylindrical chamber cut out from a poly (methyl methacrylate) (PMMA) cube with two carbon-based electrodes. The anode is a carbon brush spined around a titanium rod to improve the reaction surface and power production levels. The cathode is assembled with the membrane (membrane–electrode assembly, MEA), where carbon black and activated carbon are responsible for electron transport, a stainless steel mesh provides support and a polyvinylidene fluoride (PVDF) mixture provides impermeability. A titanium wire is, through the reactor, pressed in contact with this disk to provide electrical contact outside the reactor chamber.

The artificial wastewater composition is described in [26] and can be reduced to a mixture of a phosphate buffer with sodium, potassium, ammonia and carbon. The phosphate buffer ensures adequate pH, and the remaining elements are essential for bacterial colonies to thrive. Providing the above operation conditions are provided, MFC’s power levels are dependent on two major conditions: the substrate feeding rate and how electrodes are connected.

The substrate feeding rate sets the flow of the substrate through the reactors:

1. Continuous flow: The reactor liquid is continuously supplied with the substrate by using pumps that move it in and out. This results in low hydraulic retention (HRT), which means that the metabolic by-products are less concentrated and the energy conversion rates are lower.
2. Semi-continuous flow: The fuel in the reactor is replaced with a fixed amount at regular intervals. This leads to higher HRTs.
3. Batch or fed-batch flow: The fuel in the reactor is completely replaced when a certain parameter reaches a threshold. The parameter can be related to physical (voltage, current) or biochemical factors, such as oxidation-reduction level, pH, dissolved oxygen, and biochemical oxygen demand. This mode ensures the highest HRTs and increases energy conversion rates [26].

The biofilm conditions, which affect the power profile, depend on how the MFC is operated [27,28,29,30].

Electrode connection can also be modulated: Power production will be optimized for impedance matching and by previously imposing open-circuit conditions to the MFC. These choices can make the MFC work in four different modes:

Mode 1: Start-up—Converter start-up;
Mode 2: Stamina—For longer periods of time;
Mode 3: Balanced—Balanced power and stamina;
Mode 4: Max Energy—For maximum energy production.

Neither of these options is exclusive, and a combination of operation modes can be set. This subject will be further explored in future publications.

Maximum power levels are provided below, which are achieved with trials on a single 28 mL reactor:

Mode 1: 1.54 mW at 0.33 V (approximately 0.89 J in 20 min, 2.67 J in 4 h);
Mode 2: 1.04 mW at 0.27 V (approximately 0.23 J in 20 min, 2.7 J in 4 h);
Mode 3: 1.35 mW at 0.31 V (approximately 0.95 J in 20 min, 4.47 J in 4 h);
Mode 4: 0.58 mW at 0.24 V (approximately 0.70 J in 20 min, 8.35 J in 4 h).

Connecting individual MFCs in parallel is an interesting strategy for increasing the power levels as well as the overall extracted energy, while a series connection presents more challenges. Discussion of such strategies, as well as approaches to the regulation and storage of this energy, can be found in [31] and [32]. A specific approach to this energy’s regulation and storage will be discussed below.

### 3.2. Energy Regulation and Storage

**Figure 4:**
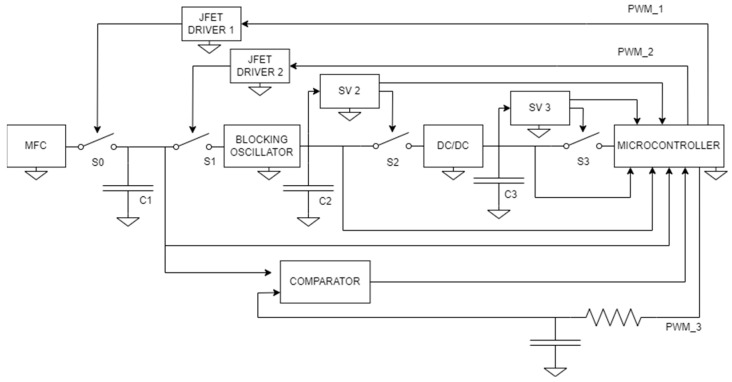
Power management system.

This subsection discusses the PMS that harvests the energy and regulates the voltage produced by the MFC. The proposed architecture, shown in Figure 4, is analyzed and discussed. The optimal point of operation, obtained from a previous experimental evaluation of the MFC, was observed when the voltage output, which depends on the applied load, was a value in the 300/400 mV range. The power management method is crucial when the main goal is to extract the maximum energy from the MFC. The proposed regulation methodology applied to harvest energy from the MFC uses different charge pumps and voltage converters to power up the MCU that rules the PMS.

The PMS has two different working stages: at first, a non-regulated starting stage harvests the necessary energy to allow the MCU to start operating. Afterward, a second regulated stage of operation starts, with the MCU regulating the energy-harvesting process. Figure 4 presents the power management schema.

#### 3.2.1. Non-Regulated Starting Stage and MCU Power-Up

The MCU needs a regulated voltage of 3.3 V to start properly. The only available energy source is the MFC. However, the output voltage of this source is low and needs proper regulation to raise it to 3.3 V.

In the non-regulated mode, the system starts up in an uncontrolled way. A capacitor C1 is placed parallel to the MFC and charged by it. On the one hand, the bigger the C1 capacity value, the slower the charging process. On the other hand, its discharge will be slow, making the capacitor assume a battery-like behavior. Capacitor C1 charge/discharge process is managed by two normally closed switches, S0 and S1. S1 and S0 are always closed in the non-regulated stage, extracting energy in continuous mode from the MFC. The function of S0 is to make the capacitor charge by connecting it to the MFC voltage source, while S1 is used to discharge C1 by connecting it with the downstream system. After the S1 switch, a blocking oscillator (BO) circuit is present [33]. It is a circuit that behaves like a charge pump, increasing the voltage on capacitor C2. Its functioning is based on positive feedback from coupling a bipolar transistor and an inverting transformer. With the positive feedback, every time the bipolar junction transistor (BJT) goes into a saturation condition, the remaining energy in the transformer core is discharged in a peak detector circuit, and the charging cycle repeats, oscillating between the saturation state of the BJT and its interdiction region, passing from its active region. This way, the output voltage undergoes a step rise every time the BJT goes into a saturation condition. Thanks to this circuit, it is possible to increase the voltage from some hundreds of millivolts up to 10 V. Downstream from the BO circuit, a DC/DC buck-boost converter is inserted. Its input voltage is in the 2–20 V range, and its output voltage is fixed at 3.3 V. This circuit regulates the BO output voltage and finally powers up the MCU.

#### 3.2.2. PMS Steady-State Operation

Once the MCU powers up, it controls the PMS. For this purpose, the following resources of the MCU are configured as follows:

- Three analog inputs;
- Three digital inputs;
- Three PWM-regulated output signals;
- V connection;
- Ground connections.

The analog inputs sense the charge voltage of the three capacitors, C1, C2, and C3, while the digital inputs indicate if a capacitor voltage is above or below a certain charge threshold to actuate the respective switches: S0 and S1 for C1, S2 for C2, and S3 for C3. In particular, S0 and S1 are implemented by two normally closed N-Junction Field Effect Transistors (N-JFETs), while S3 and S4 are normally open. In this way, it is possible to regulate the amount of charge in C1 and the discharge source, which can only be capacitor C1 (S0 open, S1 closed) or capacitor C1 and the MFC (S0 and S1 closed). SV1 and SV2 are two voltage supervisors [34] that close the S2 or S3 switches once the C2 or C3 voltage reaches a prefixed level. Also, it includes a voltage comparator, which performs a function similar to the voltage supervisors with the difference that in the voltage comparator, the reference threshold voltage is adjustable by adjusting the duty cycle of the PWM_3 voltage that goes in input to the shown resistor-capacitor (RC) filter. By considering the clock frequency of the MCU, it is possible to size the resistor and the capacitor of the filter. The bigger the resistor and the capacitor, the more negligible the pulse width modulation (PWM) effect, making the output voltage continuous. The disadvantage of bigger components can be the slow charging and discharging. However, in this application, all the signal variations are extremely slow, so the upper limit in resistance and capacitance can be considered only given by real-world constraints like the occupied area and the availability of commercial components. The comparator downstream of the filter provides the information used to decide to close or open the switches S0 and S1, which are normally closed and implemented by two N-JFETs, while S3 and S4 are normally open and implemented by two metal-oxide-semiconductor field-effect transistors (MOSFETs). In this way, it is possible to regulate the amount of charge in C1, C2, and C3 and set the PMS voltage source, which can be only capacitor C1 (S0 open, S1 closed) or capacitor C1 and the MFC (S0 and S1 closed). It is also possible to make capacitor C1 charge (S0 closed, S1 open) and power the PMS with the residual charge contained in capacitors C2 and C3. The JFETs are driven by the two shown drivers, which impose a negative voltage on the gate of the JFETs.

#### 3.2.3. JFET Driver Circuit

**Figure 5:**
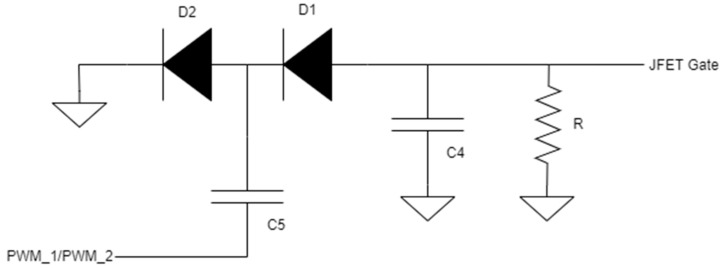
JFET driver circuit.

The circuit that produces the negative voltage is shown in Figure 5. It consists of a cascade of a high-pass filter, which makes a derivative operation to the signals PWM_1 and PWM_2, and a diode, which takes only the negative part of the signal. After the diode, D1, a capacitor, C4, and a resistor, R, are present. The resistor’s role is to pull the voltage to 0 V when diode D1 is off, while the capacitor is used to slow down the rising from the negative voltage to 0 V. In fact, with a sufficiently big capacitor and a low signal duty cycle, it is possible to drive the JFETs always open because the discharging time of capacitor C4 is longer than the time that diode D1 is off. Under these conditions, regulating the duty cycles of PWM_1 and PWM_2 signals that it is possible to maintain in an open or closed state the JFETs S0 and S1.

#### 3.2.4. Voltage Supervisors

The voltage supervisors are integrated circuits (ICs), which can give an output voltage high or low as a function of their input voltage. Differently from the comparator, the reference voltage is produced inside the device in a voltage supervisor. In function of the combination of its three selection inputs, the voltage supervisor can set a voltage threshold, above which the output signal is logically high. Otherwise, it becomes low. The main application of these circuits is the output of enabling signals or driving switching states. In this case, they are used to drive MOSFETs on and off to enable/disable the discharge of capacitors C2 and C3 to the circuits downstream. Another essential function of the voltage supervisors is to monitor the voltage of capacitors C2 and C3 and, in this way, close the connection switches with the DC/DC converter and the MCU only when the voltage reaches a proper level that is included in their normal functioning voltage range (2-20V for the DC/DC converter, 3.3 V for the MCU).

In conclusion, with the proposed PMS, it is possible, thanks to its voltage boost characteristics and big capacitors, to use this energy module to power up an MCU and, correlating to the concept of smart sensor networks, also feed several sensors/actuators which are present on a network, using a common power bus.

### 3.3. Energy Management at the Smart Sensor

The TIM is composed of five transducer channels:

1. C_1_ voltage;
2. C_2_ voltage;
3. C_3_ voltage;
4. Timeslots.
5. Proxy (C_1_ voltage, C_2_ voltage, C_3_ voltage and timeslots).

For the system configuration, the following TEDS were used:

- Meta-TEDS;
- TransducerChannel TEDS 1;
- TransducerChannel TEDS 2;
- TransducerChannel TEDS 3;
- TransducerChannel TEDS 4;
- TransducerChannel TEDS 5;
- User’s Transducer Name TEDS 1;
- User’s Transducer Name TEDS 2;
- User’s Transducer Name TEDS 3;
- User’s Transducer Name TEDS 4;
- User’s Transducer Name TEDS 5;
- PHY TEDS;
- Proposed Energy TEDS.

#### 3.3.1. Task Scheduling

Five transducer channels are configured in the meta-TEDS. The last is a proxy, so NCAP can read all the data simultaneously. When TIM starts up, it loads all the configurations contained in the TEDS and then switches to a state of lower energy consumption. It can exit this state in two ways: either through a wakeup on a port, with which the handshake with NCAP is performed, or through a wakeup on a timer, which wakes up periodically to check the reception of commands from NCAP. The periodicity with which it wakes up is defined in the time between slots variable, which is initially constant and is defined by the manufacturer in the energy TEDS but may vary depending on the decision of the energy management algorithm. The energy management algorithm periodically measures the capacitor C_3_ voltage value upon receiving a command from NCAP. These measurements identify the charging or discharging trend by adjusting the time between time slot values. TIM should return a time between slots value of zero when it knows it will not have enough energy to open a slot again. In this case, NCAP loses the handshake with this TIM.

The time slot is defined as the duration of time that the radio is on and, therefore, can receive a command. This is a constant value specified by the manufacturer in the Energy TEDS.

When the TIM is first switched on, NCAP does not know its existence; this requires a mechanism to acknowledge the new TIM: the handshake. TIM waits, in a lower power state, for the interruption of this state from a port defined for this function. When this port is activated at logic level 1, it turns on the radio and waits for commands from NCAP. The first command NCAP sends is the Query TEDS command; if it does not receive it, it sends the command again up to a maximum defined by the manufacturer; if it receives the response from TIM, it knows which TEDS it has. Knowing the existing TEDS, it will send the Read TEDS command to read each of the TEDS identified with the previous command. If everything is correct, it sends a Read Transducer Data-Set command to the proxy, knowing the values of C_1_ voltage, C_2_ voltage, C_3_ voltage and the time between slots. With this last one, it will know when TIM will listen again to receive new commands. To finish the handshake, it sends the Sleep command and counts the time for the next time slot.

**Figure 6:**
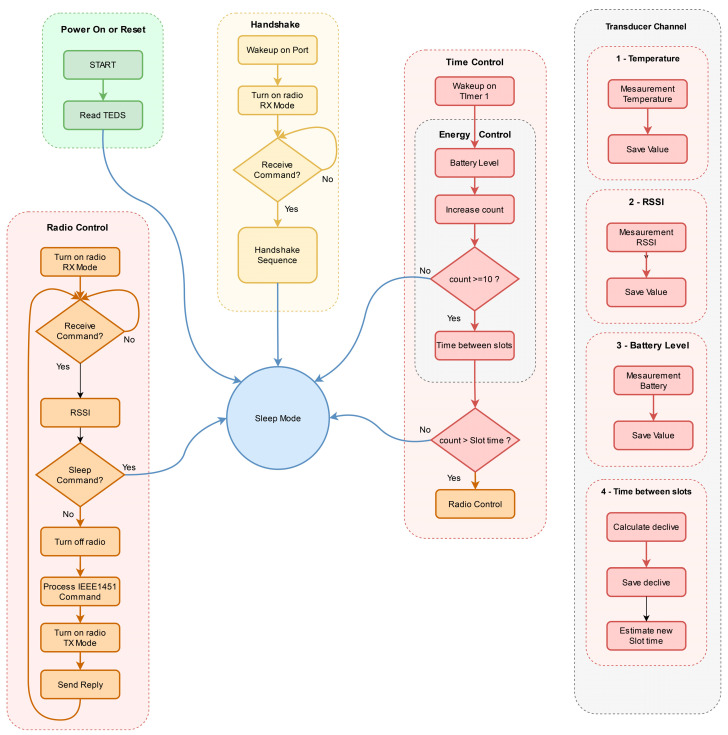
Flowchart low power operation and handshake mechanism.

Figure 6 shows the flowchart of the operation of the handshake mechanism. The TIM manufacturer shall ensure sufficient power for the handshake.

Once the handshake is completed, NCAP can send any command supported by the standard (always respecting the time slot corresponding to that TIM). In this case, it sends the Read TransducerChannel Data-Set Segment command to the transducer channel proxy. If it does not receive it, it sends the command again up to a maximum defined by the user. If it receives, it decodes and saves the received values. It sends a Sleep command and waits for the next time slot.

The transducer channels are configured with a real single precision Sample Mode representing a float 32. The temperature and Received Signal Strength Indicator (RSSI) transducer channels acquire values for an Immediate operations trigger, i.e., measured upon receipt of the Read TransducerChannels Data-Set command. The battery voltage transducer channel is measured in Continuous Sampling trigger mode, which is required for the energy algorithm to calculate the time between slots.

TIM time control, represented in the flowchart as Time Control, is the responsibility of timer 1. This is configured to interrupt the processor every second, which is a value that must be constant. It executes the available energy control algorithm every second, calculating the time between slots. This variable always has a value that is an integer multiple of the time configured in timer 1.

#### 3.3.2. Radio Management

As the amount of energy is limited and as in the IEEE 1451 standard, NCAP initiates the communication, NCAP must always know the time slot when TIM will have the radio on, as it will have to be off most of the time. As the radio is not always on, ensuring synchronization between TIM and NCAP is necessary. This requires that both know when TIM will be back in RX mode. When NCAP makes a request, it receives the sensor data and the time of the following reading on the proxy. If NCAP receives data from two different TIMs simultaneously, it discards the one from which it received the most recent data.

To better ensure time synchronization and to avoid the accumulation of errors in time counting, whenever NCAP receives a response from TIM, it sends a sleep command and restarts time counting. If synchronization is lost, NCAP marks TIM as “out of sync” and sends periodic requests until TIM responds again. TIM periodically (once a minute) measures the supercapacitor voltage and, at the end of the second measurement, calculates the slope between the last two measurements.

The slope is calculated from Equation (1) using a *U*(*t*) graph.

(1) $$U\left(t\right)=slope\times t+U_{0}$$

(2) $$slope=\frac{U\left(1\right)-U\left(0\right)}{∆t}$$

If the calculated slope value is less than zero, the TIM sends the value zero for the time between time slots if it still has enough energy, notifies NCAP that it will run out of power and enters unsynchronized TIM mode.

If the calculated slope value is equal to or greater than zero, the TIM calculates the estimated value for the time between time slots according to the following equation:

(3) $$t=\left[\left(\frac{1}{\frac{voltage\left(1\right)}{4.5}}\times offset\right)+(TITBW-offset)\right]$$

In Equation (3), the TITBW is the value defined in the energy TEDS, and the *offset* is the time constant the manufacturer must insert when manufacturing the TIM. For an *offset* value equal to 2000 and a TITBW value equal to 300, the values shown in Table 1 for the time slots were obtained using an MSP 430f2274 MCU.

**Figure 7:**
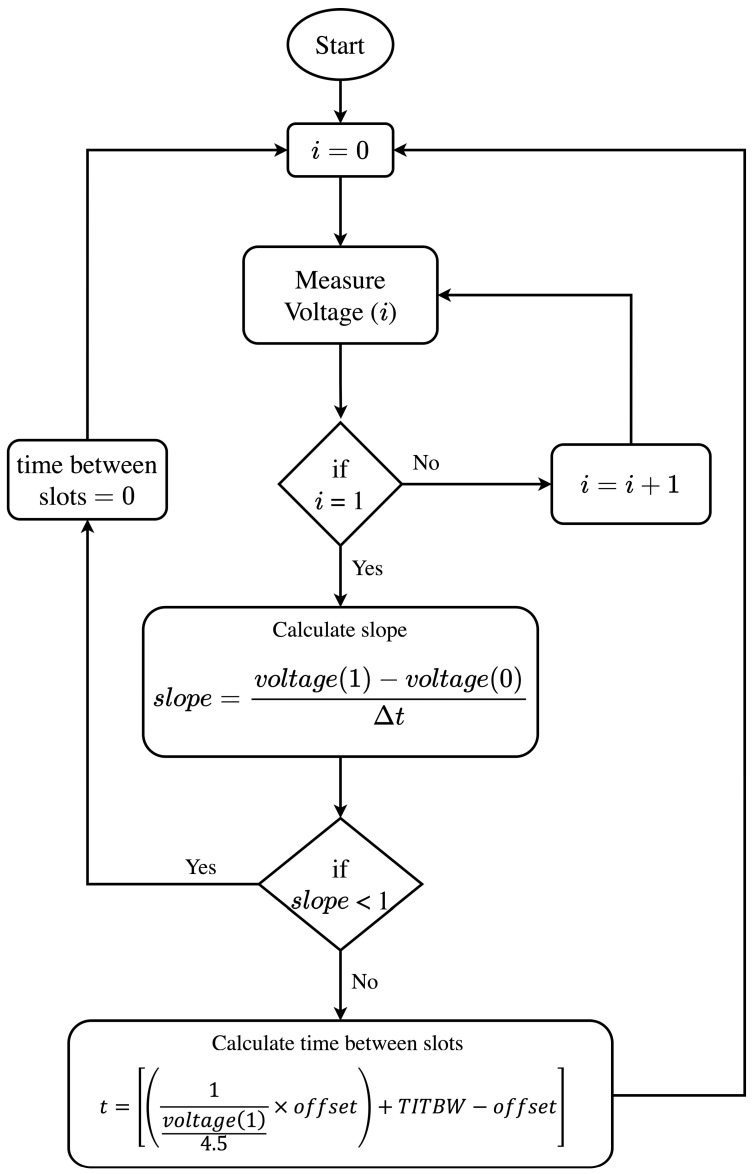
Energy control algorithm flowchart.

Figure 7 shows the organization of the algorithm that allows TIM to estimate the time it will be back in RX mode (time slots).

## 4. Security in Low-Power Sensors

Security in low-power sensors is a critical issue for the IoT, as these devices are often deployed in various environments and applications that require data protection and integrity. However, low-power sensors have limited resources, e.g., battery life, processing power, and memory, making them vulnerable to attacks and compromising functionality. These sensors often need to perform security operations, such as encryption, authentication, and integrity checking, which consume a significant amount of energy and reduce their lifetime. Therefore, it is essential to design and implement efficient and effective security solutions for low-power sensors that can balance the trade-off between security and energy [35]. The security challenges of using low-power sensors in IoT applications are mainly related to protecting data and devices from various attacks and threats [36].

Energy harvesting for data transmission has been used in different approaches. Fang et al. use capacitors to acquire and store wireless energy and use this energy to provide security by employing cryptography algorithms after transmitting the data [35]. Alimi et al. [36] wrote a survey about the low power area network (LPWAN). The authors analyze the key security requirements and threats for LPWANs, such as confidentiality, integrity, availability, authentication, authorization, and non-repudiation. It also discusses the common types of attacks that can target LPWANs, such as replay attacks, denial-of-service attacks, wormhole attacks, and eavesdropping attacks. The paper presents the existing security solutions and protocols for LPWANs, such as encryption, digital signatures, key management, access control, and intrusion detection. It also compares the advantages and disadvantages of different security approaches for LPWANs, such as symmetric cryptography, asymmetric cryptography, and lightweight cryptography. The security challenges include the following [36]:

- Data confidentiality: Low-power sensors often transmit sensitive data over wireless channels, which can be intercepted by malicious attackers. To protect the data from eavesdropping, encryption techniques are needed. However, encryption algorithms usually require high computational power and memory, which are scarce resources for low-power sensors. Therefore, finding efficient and lightweight encryption schemes is challenging for low-power sensor security.
- Data integrity: Low-power sensors can be vulnerable to data tampering or injection attacks, where an attacker modifies or inserts false data into the sensor network. This can compromise the accuracy and reliability of the sensor data and affect the decision making of IoT applications. To ensure data integrity, authentication and verification techniques are needed. However, these techniques impose additional overhead on low-power sensors, such as extra communication and computation costs.
- Data availability: Low-power sensors rely on batteries or energy harvesting to operate. It can be subject to denial-of-service (DoS) attacks, where an attacker exhausts the sensor’s energy or bandwidth by sending excessive requests or packets. This can prevent the sensor from performing normal functions and delivering data to the IoT applications. To prevent DoS attacks, power management and resource allocation techniques are needed. However, these techniques must also balance performance and energy efficiency trade-offs.

Panoff et al. propose a lexicon to distinguish types and methods of attacks on sensors based on the stage of the targeted sensor data processing pipeline, the type of signal that is manipulated, and the type of device used to perform the attack. The attacks are classified as classical, data, sensor exploit, algorithmic attacks, sensor commandeering, and signal. The paper also provides examples of each attack category and their potential impacts on sensor security. The existing defenses against sensor attacks include encryption, digital signatures, key management, access control, and intrusion detection. The paper also compares the advantages and disadvantages of different security approaches for sensors, such as symmetric cryptography, asymmetric cryptography, and lightweight cryptography. The research gaps and future directions for improving the security of sensors include developing more efficient and robust security mechanisms, addressing the trade-off between security and performance, enhancing the interoperability and scalability of sensors, and ensuring the privacy and trust of users and devices [37].

Todeschi et al. wrote a survey on network security issues in energy-harvesting (EH) networks employing energy sources, e.g., radio frequency, solar, mechanical, wind, and thermoelectric. It analyzes the key security requirements and threats to EH networks, such as confidentiality, integrity, availability, authentication, authorization, and non-repudiation. It also discusses the common types of attacks that can target EH networks, such as replay attacks, denial-of-service attacks, wormhole attacks, and eavesdropping attacks. The paper presents the existing security solutions and protocols for EH networks, such as encryption, digital signatures, key management, access control, and intrusion detection. It also compares the advantages and disadvantages of different security approaches for EH networks, such as symmetric cryptography, asymmetric cryptography, and lightweight cryptography. The paper identifies the research gaps and future directions for improving the security of EH networks, such as developing more efficient and robust security mechanisms, addressing the trade-off between security and performance, enhancing the interoperability and scalability of EH networks, and ensuring the privacy and trust of users and devices, the attacks are as follows [38]:

- Replay attacks are when an attacker captures and retransmits a valid message or data packet to the receiver, pretending to be the original sender. This can cause confusion, duplication, or deception in the communication between the sender and the receiver. Replay attacks can affect the security and performance of low-power sensor networks, which are widely used in IoT applications [39].
- Eavesdropping occurs when an attacker intercepts and listens to the communication between two or more parties without their knowledge or consent. This can compromise the confidentiality and privacy of the transmitted data, such as sensor measurements, location, or identity.
- Spoofing is when an attacker impersonates another entity, such as a sensor node, a base station, or a user, by forging its identity or credentials. This can undermine the authentication and authorization mechanisms of the system and allow the attacker to gain unauthorized access or privileges.
- Man-in-the-middle attack is when an attacker inserts itself between two communicating parties and relays or modifies their messages. This can violate the integrity and authenticity of the transmitted data and enable the attacker to manipulate or alter the communication.
- DoS is when an attacker prevents or disrupts a service or resource’s normal functioning or availability by overwhelming it with excessive requests or traffic. This can degrade the system’s performance and quality of service and render it unusable or inaccessible.

Dhunna and Al-Anbagi [39] proposed a low-power cyber-security mechanism for smart grid monitoring applications for wireless sensor networks (WSNs). The paper analyzes the security requirements and challenges of WSNs in smart grid environments, such as confidentiality, integrity, availability, authentication, authorization, and non-repudiation. It also identifies the common attacks that can target WSNs in smart grids, such as denial of sleep, forge, and replay attacks. Denial of sleep attacks aims to drain the battery power of the sensor nodes by preventing them from entering sleep mode. Forge attacks aim to inject false or malicious data into the network by impersonating legitimate nodes. The paper presents a novel security mechanism that can detect and isolate these attacks energy efficiently. The mechanism comprises three components: a lightweight encryption scheme, a dynamic key management scheme, and a distributed intrusion detection scheme. The lightweight encryption scheme uses a combination of symmetric and asymmetric cryptography to protect the data in transit and at rest. The dynamic key management scheme uses a hierarchical structure to periodically generate, distribute, and update cryptographic keys. The distributed intrusion detection scheme uses a cooperative approach to monitor and analyze the network behavior and traffic and alert the network manager if any anomalies or deviations are detected. The paper evaluates the performance of the proposed security mechanism using simulations and compares it with existing techniques. It uses the MATLAB and NS2 simulator combined with data from Chipcon CC2420 radio and Atmega128L MCU. The results show that the proposed mechanism can achieve high security levels while maintaining low power consumption, constant delay, and high reliability. There are several ways to prevent replay attacks in low-power sensor networks:

- Encryption and authentication are techniques that protect the data from eavesdropping and tampering by hackers. Encryption scrambles the data being transmitted, making it unreadable to anyone who does not have the key to decrypt it. Authentication verifies the identity and validity of the sender and the receiver, ensuring they are who they claim to be. However, encryption and authentication algorithms usually require high computational power and memory, which are scarce resources for low-power sensors. They also increase the size of the data packets, which can consume more energy for transmission and reception. Therefore, finding efficient and lightweight encryption and authentication schemes is challenging for low-power sensor network security [36,39].
- Session keys or one-time passwords are only valid for one transaction or communication session and cannot be used again. They can prevent replay attacks by ensuring each message has a unique and random code different from the previous or next one. If an attacker tries to replay a message with an expired or invalid code, the receiver will reject it. However, session keys or one-time passwords also require additional communication and synchronization between the sender and the receiver, which can increase the energy consumption and latency of the network [36,39].
- Timestamps or sequence numbers are pieces of information that indicate the order or time when a message was created or sent. They can prevent replay attacks by limiting a message’s validity period or range so that it cannot be resent after a certain time interval or sequence number. If an attacker tries to replay a message with an outdated or out-of-order timestamp or sequence number, the receiver will ignore it. However, timestamps or sequence numbers also depend on the accuracy and synchronization of the clocks or counters of the sensors, which can be affected by environmental factors or malicious manipulation [39].
- Using encryption and authentication for low-power sensor networks is a way to enhance the security and privacy of the data transmitted by the sensors. However, encryption and authentication also present some challenges for low-power sensor networks, such as algorithms, key management, and interoperability.
- Key management is the process of generating, distributing, storing, updating, and revoking cryptographic keys. It is essential for ensuring the security and functionality of encryption and authentication. However, key management is also complex and challenging for low-power sensor networks due to their large scale, dynamic topology, resource constraints, and attack vulnerability. Therefore, designing scalable and robust key management protocols is challenging for low-power sensor network security [40].
- Interoperability or compatibility allows different devices or systems to work together without conflicts or errors. It is important to ensure the interoperability and usability of encryption and authentication. However, compatibility is also a difficult issue for low-power sensor networks due to their heterogeneity, diversity, and evolution. Different sensors may have different hardware, software, or standards, which can affect their encryption and authentication capabilities or requirements. Therefore, achieving compatibility and harmonization among sensors is challenging for low-power sensor network security [41].

Public-key and secret-key cryptography are two types of encryption systems that use different kinds of keys to encrypt and decrypt data. Encryption transforms data into a form that is unreadable by anyone who does not have the appropriate key. Decryption is the reverse process of recovering the original data from the encrypted form. The purpose of encryption is to ensure the privacy and security of data, especially when it is transmitted over insecure channels. A key is an information or parameter used to perform encryption and decryption. In secret-key cryptography, also known as symmetric cryptography, the same key is employed for encrypting and decrypting. The sender and the receiver share exactly the same secret key to secure the communication. The advantage of secret-key cryptography is that it is fast and efficient, but the disadvantage is that it requires a secure way to distribute the key to both parties [42].

Some examples of algorithms for cryptography and authentication in low-power sensor networks follow:

### 4.1. Symmetric Cipher

Symmetric ciphers require a common pre-set secret key between the two agents during the communication to encrypt and decrypt the data. Five block ciphers have been developed until today. It is classified by its internal structure, e.g., substitution permutation networks (SPN), Feistel networks, add–rotate–XOR (ARX), NSLFSR-based and hybrid. SPN uses sequential subtraction and permutation boxes to prepare the data for the next round. The Feistel network structure is a common way to build block ciphers. A network consists of several rounds of processing, where each round splits the input block into two halves and applies a non-linear function (called S-box) to one half using a subkey derived from the main key. The function’s output is then combined with the other half using an exclusive–or (XOR) operation, and the two halves are swapped for the next round. The S-box and the permutation are designed to provide good diffusion and confusion properties and hardware optimization [43].

Advanced Encryption Standard (AES) is an SPN block cipher that uses a 128, 192, or 256-bit key to encrypt and decrypt 128-bit data blocks. It is one of the most widely used algorithms and was standardized by NIST. AES is secure, efficient, and flexible and can be implemented in hardware or software. However, AES also requires high computational power and memory, which can be challenging for low-power sensors [44]. AES may be suitable for low-power energy-harvesting sensors that need strong and versatile encryption and compatibility with existing standards and devices. However, AES also needs additional mechanisms for authentication and key management, which may increase the complexity and cost of the system [45].

Data Encryption Standard (DES) is a Feistel encryption method widely used in the past but is now considered insecure due to its short 56-bit key length. DES energy harvesting is based on the observation that the DES algorithm involves many permutations and substitutions of bits, which can be implemented using reversible logic gates. Reversible logic gates are circuits that do not dissipate energy during their operation and can recover some energy from their inputs [46].

PRESENT is an NPN cipher with a block size of 64 bits and a key size of either 80 or 128 bits [47].

CLEFIA is a Feistel block cipher algorithm developed by Sony in 2007. It is a type of encryption method that uses a secret key to transform a fixed-size block of data into another block of data that is hard to decipher without the key. CLEFIA has a block size of 128 bits and a key size of 128, 192, or 256 bits. It uses a generalized CLEFIA with 18, 22, or 26 rounds, depending on the key size [48].

A lightweight encryption algorithm (LEA) cipher is a 128-bit ARX block with a 128, 192, or 256-bit key size. It uses a simple structure of modular addition, bitwise rotation, and bitwise XOR operations, which are efficient on common processors. PRESENT, CLEFIA and LEA are standardized in the ISO/IEC 29192-2:2019 Information security—Lightweight cryptography—Part 2: Block ciphers [49].

PRINCE is an SPN cipher with a block size of 64 bits and a 128-bit key size. The key is split into two parts of 64 bits each and extended to 192 bits by a mapping function. The input is XORed with the first part and then processed by a core function using the second. The output of the core function consists of 11 rounds, each involving a round constant, a non-linear layer, and a linear layer with encryption and decryption very similar except for the round constants and the key order [50].

Midori is an SPN lightweight block cipher developed by Sony in 2015. It is designed to provide fast and secure encryption for low-energy devices, such as medical implants, sensor networks, and IoT devices. Midori cipher has two versions: Midori64 and Midori128, which have block sizes of 64 and 128 bits, respectively. Both versions use a 128-bit key and a simple structure of modular addition, bitwise rotation, and bitwise XOR operations [51].

KATAN is an NLFSR-based block cipher designed for low-end devices, such as RFID tags, with limited resources and the need to encrypt and decrypt data securely. KATAN cipher is part of a family of six block ciphers, which is divided into two flavors: KATAN and KTANTAN. All block ciphers in this family use an 80-bit key and have different block sizes: 32, 48, or 64-bit. KATAN cipher has 18, 22, or 26 rounds, depending on the block size [52].

PICCOLO cipher is a Feistel lightweight block cipher developed by Sony in 2011. This cipher has a block size of 64 bits and either an 80 or 128-bit key size [53].

Secure IoT (SIT) cipher is a lightweight encryption algorithm developed for secure IoT applications. It is a symmetric key block cipher that uses a 64-bit key and a 64-bit block size. It is based on a mixture of Feistel and SPN structures, which provide good security and efficiency. SIT cipher has been implemented on a low-cost MCU compared with other lightweight ciphers, such as LEA, PRESENT, and PICCOLO [54].

LBLOCK cipher has a Feistel block size of 64 bits and a key size of 80 bits, with 32 rounds of processing [55].

### 4.2. Stream Cipher

A stream cipher is an encryption method that uses a secret key and a nonce (a random number used only once) to generate a pseudo-random stream of bits combined with the plaintext using the XOR operation to produce the ciphertext. Stream ciphers are usually fast, simple, and flexible. It requires careful management of the keys and nonces to avoid reusing them, which can compromise the security of the encryption. Stream ciphers can also be vulnerable to certain types of attacks, such as bit-flipping or replay attacks, if not combined with authentication mechanisms.

Grain 128AEADv2: It improved the previous Grain 128 cipher with an internal state size of 256 bits, consisting of two 128-bit registers: a non-linear feedback shift register (NLFSR) and linear feedback shift register (LFSR). The cipher uses a 128-bit key and a 96-bit nonce to initialize the registers and then performs 256 clocking rounds to warm up the state. The output stream is generated using a Boolean function to the state bits and a subkey derived from the key. It supports variable tag lengths up to 32 bits for message authentication [56].

ChaCha20 was derived from the Salsa cipher and uses a 256-bit key and a 64 or 96-bit nonce to generate a pseudo-random stream of bits, which are combined with the plaintext using the XOR operation to produce the ciphertext [57,58].

### 4.3. Asymmetric Cipher/Public-Key Cipher

The Diffie–Hellman (DH) cipher is based on a mathematical method of public-key cryptography, allowing two parties to establish a shared secret key over an insecure channel without revealing it to anyone else. This key encrypts and decrypts their communications using a key cipher. The algorithm requires both the sender and receiver to have a pair of keys (private and public). Computing the public-key with the private-key of the other parts can find the same shared secret number. This number encrypts and decrypts the message data [59].

Rivest–Shamir–Adleman (RSA) cryptography is a method of encrypting and decrypting data using public-key and private-key pairs. It is based on the mathematical problem of factoring large numbers, which is considered hard to solve. It was inspired by the Diffie–Hellman algorithm [60].

ECC is a public-key cryptography scheme that uses mathematical curves to generate public and private keys. ECC can provide the same level of security as RSA with much smaller key sizes, which can reduce the communication and computation costs for low-power sensors. ECC can also support various cryptographic functions, such as encryption, digital signatures, key exchange, or identity-based encryption. ECC may be suitable for low-power energy-harvesting sensors that need asymmetric encryption and authentication as well as scalability and flexibility. However, ECC also involves complex mathematical operations, which can be difficult to implement on resource-constrained devices [61].

Ed25519 is a digital signature algorithm that uses ECC to generate and verify signatures. It is based on the Curve25519 curve, which is designed for high performance and security. Ed25519 is widely used in various applications, such as SSH, TLS, Tor, and cryptocurrencies [62]. MCU implementation versions of Ed25519 can run on small and low-power devices, such as embedded systems, IoT devices, or smart cards, and they can optimize the speed, memory, and energy consumption while maintaining security and functionality.

Dhanda et al. wrote a survey of available lightweight cryptographic primitives. It analyzed 54 protection techniques, e.g., lightweight block ciphers, stream ciphers, hash functions, and ECC, comparing metrics such as energy and power, hardware and software efficiency, and figure of merit (FoM). The hardware used was the 8051, ATtiny 45, and ATmega128 MCUs. It concludes that AES and ECC are the most suitable lightweight cryptographic for IoT devices. However, ECC needs to be improved to become the first choice. It consumes more memory for its operations [63]. In [64], Prakasam et al. proposed and implemented a hybrid lightweight cryptography authentication scheme (HLCAS) that utilizes the 8-bit manipulation principle. The HLCAS combines the advantages of symmetric and asymmetric cryptography to achieve low latency, area and optimal power consumption. The HLCAS is verified and validated for speech signals using MATLAB.

Zakaria et al. wrote a systematic review of the lightweight block cipher. The authors studied 101 algorithms, focusing on their security. The author concluded that the PRESENT is the most used lightweight block cipher, being secure from key attacks. The second most popular is the AES, which is followed by PRINCE and Midori. Also, to develop a secure, lightweight block cipher, combining substation and permutation is the best solution, providing confusion and diffusion properties [65].

## 5. Discussion

This section will present the surveys and paper that compare the algorithms for 8, 16, and 32-bit MCUs to evaluate if the energy generated by the MFC can power the radio while also providing enough energy to encrypt and authenticate a message using the IEEE 1451 family of standards.

The first step involves choosing a suitable cryptographic algorithm. Cryptographic algorithms have different energy consumption profiles depending on their key size, block size, number of rounds, mathematical operations, and hardware or software implementation. An algorithm needs to be chosen that meets security requirements but also minimizes the energy consumption of sensors.

Encryption and authentication are complementary techniques that protect your data from eavesdropping and tampering. However, using them separately can increase the energy consumption of your sensors, as you need to perform two operations and send two messages for each data packet [66].

Some algorithms perform the encryption and authentication together in one algorithm, such as ChaCha20-Poly1305, which can provide confidentiality and integrity in one operation and message. This can reduce the energy consumption of sensors by avoiding duplication and overhead.

The second step involves adapting to the network conditions. The energy consumption by sensors using cryptography can also depend on the network conditions, such as the channel quality, the traffic load, the node density, or the node mobility. The cryptographic parameters or protocols to the network conditions optimize the energy consumption of the sensors. For example, adaptive key management schemes can be used to adjust the key length or frequency according to the network security level or energy availability.

The authors in [67] compared the energy consumption of TEA, XTEA, and SKYPJACK for the 8-bit MCU PIC18F45K22 and ST-TX03-ASK 434 MHz radio module. XTEA had the best energetic performance for encrypting the data. Prakasam et al. [68] tested the energy-efficiency cryptography algorithms using Sparten3E XC3S500E Field Programmable Gate Arrays (FPGAs) operating in 8-bit operation. The algorithms tested were AES, DES, CLEFIA, PRESENT, KATAN, and SIT and their new proposal, the enhanced energy-efficient lightweight cryptography method (E3LCM). As a result, the new proposal using a 64-bit key size consumes 202 mW, followed by SIT with the same key size, which consumes 221 mW, while KATAN using the 80-bit key size consumes 234 mW with a 128-bit key size, and PRESENT consumes 240 mW, which is followed by the CLEFIA, DES, and AES with the same key size.

Di Mauro et al. implemented an adaptive new version of on-demand medium access control (ODMAC) for an energy-harvesting wireless sensor network using an eZ430 16-bit board from TI. Energy consumption data were absent from this paper [69]. Schaumont et al. provided a study of the authentication algorithms using a 16-bit MCU MSP430 and RF 2500 radio from TI. The tests were made using the algorithms hash-based message authentication code (HMAC), the elliptic curve digital signature algorithm (ECDSA), Lamport–Diffie one-time-signature (LD-OTS), and Winternitz one-time signature (W-OTS). The results showed that the algorithms based on MAC were lighter compared to ECDSA algorithms with energy ratios of 1.21 mJ and 6.43 mJ, respectively [70]. Hass et al. [71] compared the energy efficiency of TinyECC software with the VaulIC420 hardware. The AES and ECDSA algorithms were tested, and the results presented that the software version consumes less energy for 16-byte vectors from 1.267 to 0.123 mJ. Symmetric algorithms, such as AES, were more efficient with the software. However, the hardware was 76% more efficient when it used the ECDSA signature. Suslowicz et al. tested the energy harvesting in sensors operating during small periods. The MSP 430 16-bit Reduced Instruction Set Computer (RISC) and MSP 432 32-bit ARM MCUs were analyzed by implementing the AES in Counter mode (AES-CTR) algorithm. The MSP430 consumes 244.4 μJ in software implementation, whereas hardware consumes 17.8 μJ to run the AES-CTR. The MSP 432 consumes 384 μJ software and 44.6 μJ hardware [72]. The authors in [73] compared an implementation of Trivium stream cipher in two MCUs, the MSP 430 and the ATmega128L. The results showed that using the Trivium cipher with a precomputed key reduced energy consumption by 14%, and Trivium implemented in the MPS 430 performs better than the AES algorithm.

Aslan et al. [6] compared the energy consumption between the PRESENT, CLEFIA, PICCOLO, PRINCE, and LBLOCK algorithms employing an MSP 430FR6994 TI board. The authors concluded that the LBLOCK had the lowest energy consumption, spending 5.812 mJ in 10 s. Hatzivasilis et al. [43] wrote a review of the lightweight block ciphers, evaluating 52 block ciphers in software and hardware implementations. The hardware analyzed uses CMOS technology. The results showed that the best algorithm or 16-bit MCU was HB-2, which was followed by HB, AES, Fantomas, Robin, SPECK, Klein, HIGHT, TEA, XTEA, GOST, SIMON, LBLOCK, PRINCE, PICCOLO, TWINE, DESXL, NOEKEON, IDEA, PICCOLO, SEA, MIBS, PRINTCip, CLEFIA, SIMON, mCrypton, PRESENT, KATAN, and KTANTAN. Considering more metrics, such as ROM, RAM, latency, energy, and throughput, the best algorithms for 16-bit are Fantomas-128, Robin-128, SPECH-96, AES-128, PRINCE-128, DESXL-184, SEA-96, and PRESENT-80. The authors [74] tested the information leaking in low-power sensors and proposed a new approach using adaptive group encoding (AGE) to protect communication between the devices. The new method was tested in the TI MSP430 MCU, consuming 0.154 mJ for encoding a message. Mickaël et al. developed a survey with 21 block ciphers employing an MSP430 MCU. The author concluded that block ciphers such as SEA or SPECK had a good performance, whereas hardware ciphers such as LED, PRESENT, KATAN, and KTANTAN had a better performance by their bit-oriented nature [75].

Kao et al. compared the ChaCha20–Poly1305 algorithms that implement authenticated encryption with associated data (AEAD) with the AES-GCM using a Zedboard FPGA. The author estimated that the energy consumption for 50 bytes of data employing the ChaCha20-Poly1305 was 7 μW, whereas the energy wasted by the AES-GCM was 27 μW [76]. In [77], the authors compared the Chacha20 and AES algorithms in 32-bit MCU to prove attacks, concluding that the remote application ChaCha20 is a better choice. However, in side channels, such as the Controller Area Network (CAN) network, the AES is better. De Santi et al. [57,58] compare the ChaCha20-Poly1305 with AES128-GCM, AES128-EAX, AES128-CCM, and NORX32 in a 32-bit MCU for AEAD and conclude that ChaCha20–Poly1305 consumes less runtime cycles and has a small size compared with AES implementations. ChaCha20–Poly1305 also has the best energy performance when compared with the ciphers AES, Rivest Cipher 6 (RC6), Twofish, SPECK128 and LEA using the mobile CPUs ARMv7-a and ARMv8-a [78].

This discussion will be driven by the MSP 430 16-bit MCU family employed in previous works by the authors in previous papers [3,9,10] about sensor communication without security mechanisms provided. In a previous paper [79], the authors analyzed the energetic consumption of the ez430-RF2550 with MSP430F2274 MCU in LPM0 mode connected to a TI 2500, implementing an IEEE 1451 TIM and an ez430-RF2550 connected with a Raspberry Pi 3b+ as an IEEE 1451 NCAP. Three tests were made. First, the communication occurs every second, consuming 63.7 mJ in total (reception, transmission, processing and LPM0). Second, waiting for a command from the NCAP to the TIM resulted in an energy consumption of 27 mJ. The third one uses the embedded energy management control group (EEMCG). Using the information from the resources provided by the IEEE 1451.0 standards, the energy consumption in total was reduced to 6.63 mJ.

Currently, the IEEE 1451 family of standards does not provide a mechanism to natively secure the TIM and the NCAP communication. Mitterer et al. [80] proposed a secure TEDS that contains information about the key exchange and permission management to provide security and trust. The algorithms supported are RSA, DSA, ECDSA, and ElGamal.

**Figure 8:**
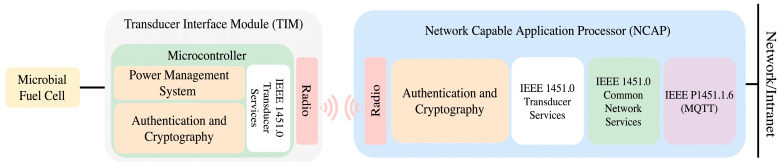
Wireless message exchange from NCAP to TIM powered by MFC.

Figure 8 shows the internal structure of network elements of an IEEE 1451 infrastructure. This scenario evaluates energy consumption during secure communication between sensors in the IoT world.

The IEEE 1451 family of standards presented in Section 2 can use the energy provided by the MFC in Section 3.1, while the low-power energy management provided in Section 3.2 and Section 3.3 can secure the communications between sensors by implementing some of the cryptography and authentication algorithms presented in Section 4 in low-power 8, 16, and 32-bit MCUs.

The IEEE 1451.0-2007 standard defines a command for a request from the NCAP to the TIM and commands an answer from the TIM to the NCAP. The command from the NCAP is a message structure defined by the IEEE 1451.0 standard and has nine octets or 72 bits. The answer from NCAP to the TIM can have different values: for example, seven octets or 56 bits for a Read TransducerChannel Data-Set Segment command. It concludes that the energy generated by MFC with appropriated energy management can be used to secure communication by employing encryption, authentication or both simultaneously. It employed the energy consumption (in μJ/bit) for the best implementation for the 16-bit MCU, obtained from Hatzivasilis et al. [43] and used the energy measured by the authors in [79] that calculates the communication using the ez430-RF2550 MCU with radio 6.63 mJ. The NCAP command will consume x μJ/bit × 72 for the request command and x μJ/bit × 56 for the response command, increasing both results plus more with 6.63 mJ from the radio. The MFC using mode 3 from Section 3.1 can provide approximately 950 mJ in 20 min, meaning the system can be powered for a certain period of time. A Read TransducerChannel Data-Set Segment command for request and response containing the energy consumption per bit from the authors in [43] and the estimated percentage for the request and response with the total percentage of energy consumption for the encrypted communication of a TIM are presented in Table 2.

Based on Table 2, the HB-2 and AES algorithms have good performance for the low-power MCU. In contrast, KTANTAN is not recommended, while PRESENT, KATAN, and LED consume more energy. Also, the ECC does have the worst performance compared with AES: “On the basis of execution time, AES is 100–1000 times faster than ECC on 8-bit microcontrollers. Execution time can be improved by reducing the computational complexity of the algorithm” [63]. For encryption and authentication, the ChaCha20–Poly1305 performs well and can be tested [81,82]. The energy consumption for ChaCha20–Poly1305 using the 8-bit MCU to process and transmit a 2400-bit message consumed 131.2 μJ, while the AES consumed 241.7 μJ [81]. Using a 32-bit MCU, the authors in [82] consumed 1.54 μJ to transmit a 256-bit message wirelessly.

An MFC can power an IoT sensor with security. Some challenges need to be addressed, such as managing the energy generated by the energy-harvesting process and testing the algorithms for cryptography, authentication, and both cryptography and authentication to determine the better choice for environmental monitoring based on the key size, block size, algorithm cycles, and energy consumption employing 8, 16, 32-bit MCUs.

## 6. Conclusions

With the work developed and presented, it was demonstrated that it is possible to approach the development of a sensor network considering several factors. Thus, the developed sensor uses the chemical energy contained in wastewater through degradation by bacteria in a biological reactor. As noted, the energy produced is scarce.

The discussion presented in this paper aims to provide a clear vision of how to integrate energy harvesting with security. It concluded that the energy generated by the MFC could be used to encrypt and authenticate the sensor that can be used in different environments, such as the monitoring environment. The IEEE 1451 family of standards does not support a native security mechanism at the TIM. However, it has been shown that a TIM can be powered and securely transmit the data wired or wireless to the local network to the intranet.

The future work will test different implementations of the cryptography and authentication algorithm in various scenarios, such as when a sensor only needs cryptography, only when authentication is needed, and also when cryptography and authentication in both cases are needed. The remaining work will be carried out in low-power MCUs and FPGAs to determine how to create a secure sensor network using the IEEE 1451 family of standards.

## Figures and Tables

**Table 1:** Correlating voltage and time between time slots.

Voltage (V)	Time (s)
3.6	800
3.7	732
3.8	668
3.9	608
4	550
4.1	495
4.2	443
4.3	393
4.4	345
4.5	300

**Table 2:** Read TransducerChannel Data-Set Segment command employing the ciphers.

Cipher	Key Size (bits)	Energy Consumption (μJ/bit)	Energy Consumption Request (mJ)	Energy Consumption Response (mJ)	Energy Consumption Request (%)	Energy Consumption Response (%)	Energy Consumption Total (%)
GOST	256	13.8	7.6236	7.4028	0.80%	0.78%	1.58%
HB	256	1.6	6.7452	6.7196	0.71%	0.71%	1.42%
AES	128	3.8	6.9036	6.8428	0.73%	0.72%	1.45%
PRINCE	128	34.2	9.0924	8.5452	0.96%	0.90%	1.86%
Robin	128	6.6	7.1052	6.9996	0.75%	0.74%	1.48%
Fantomas	128	4.9	6.9828	6.9044	0.74%	0.73%	1.46%
TEA	128	11.8	7.4796	7.2908	0.79%	0.77%	1.55%
XTEA	128	12.5	7.530	7.330	0.79%	0.77%	1.56%
CLEFIA	128	132.4	16.1628	14.0444	1.70%	1.48%	3.18%
Piccolo	128	49.2	10.1724	9.3852	1.07%	0.99%	2.06%
HB	128	6.2	7.0764	6.9772	0.74%	0.73%	1.48%
HB-2	128	0.2	6.6444	6.6412	0.70%	0.70%	1.40%
mCrypton	96	146.4	17.1708	14.8284	1.81%	1.56%	3.37%
SEA	96	59.5	1.0914	9.962	1.15%	1.05%	2.20%
SIMON	96	17.4	7.8828	7.6044	0.83%	0.80%	1.63%
PRESENT	80	1.268	97.9476	77.6548	10.31%	8.17%	18.48%
LED	80	939.9	74.3028	59.2644	7.82%	6.24%	14.06%
LBLOCK	80	20.5	8.106	7.778	0.85%	0.82%	1.67%
Piccolo	80	28.9	8.7108	8.2484	0.92%	0.87%	1.79%
KATAN	80	1.004	78.9684	62.8932	8.31%	6.62%	14.93%
KTANTAN	80	13.814	1001.2956	780.2588	105.40%	82.13%	187.53%
mCrypton	64	145.5	17.106	14.778	1.80%	1.56%	3.36%
LED	64	155	17.790	15.310	1.87%	1.61%	3.48%

## Data Availability

Not applicable.

## Abbreviations

The following abbreviations are used in this manuscript:

AEAD	Authentication encryption with associated data
AES	Advanced encryption standard
AES-CRT	AES counter mode
AGE	Adaptive group encoding
BJT	Bipolar junction transistor
BO	Blocking oscillator
CAN	Controller area network
DES	Data encryption standard
DH	Diffie–Hellman
DoS	Denial of service
E^3^LCM	Enhanced energy-efficient lightweight cryptography method
ECC	Elliptic curve cryptography
ECDSA	Elliptic curve digital signature algorithm
EH	Energy harvesting
EEMCG	Embedded energy management control group
FPGA	Field Programmable Gate Array
FoM	Figure of merit
LEA	Lightweight encryption algorithm
LFSR	Linear feedback shift register
LPWAN	Low power area network
HLCAS	Hybrid lightweight cryptography authentication scheme
HMAC	Hash-based message authentication code
IoT	Internet of things
IEEE	Institute of Electrical and Electronics Engineers
MAC	Media access control
MEA	Membrane electrode assembly
MCU	Microcontroller unit
MFC	Microbial fuel cell
mJ	Millijoule
MOSFET	Metal oxide semiconductor field effect transistor
MQTT	Message queuing telemetry transport
mW	Milliwatt
NCAP	Network Capable Application Processor
NIST	National institute of standards and technology
NLFSR	Non-linear feedback shift register
oDMAC	On-demand medium access control
PMS	Power Management System
PWM	Pulse width modulation
PVDF	Polyvinylidene fluoride
RISC	Reduced instruction set computer
RSA	Rivest–Shamir–Adleman
RSSI	Received Signal Strength Indicator
SIT	Secure IoT
SPN	Substitution permutation network
TEDS	Transducer electronic data sheet
TI	Texas Instruments
TIM	Transducer interface module
TIM	Transducer interface module
WSN	Wireless sensor network
XOR	Exclusive-or
μW	Microwatt
μJ	Microjoule
